# 奥妥珠单抗治疗免疫性血栓性血小板减少性紫癜1例报告并文献复习

**DOI:** 10.3760/cma.j.cn121090-20241123-00474

**Published:** 2024-12

**Authors:** 晓彤 陈, 艳秋 赵, 琪 李, 海涛 李, 丹丹 李, 倩 张, 金月 付, 冬雪 卢, 东阳 张, 圣瑾 范

**Affiliations:** 哈尔滨医科大学附属第一医院血液科，哈尔滨 150070 Department of Haematology, The First Affiliated Hospital of Harbin Medical University, Harbin 150070, China

**Keywords:** 免疫性血栓性血小板减少性紫癜, 奥妥珠单抗, 血管性血友病因子裂解蛋白酶, Immune thrombotic thrombocytopenic purpura, Obinutuzumab, ADAMTS13

## Abstract

**目的:**

探讨奥妥珠单抗治疗免疫性血栓性血小板减少性紫癜（iTTP）的安全性和有效性。

**方法:**

回顾哈尔滨医科大学附属第一院血液科收治的1例iTTP患者的诊疗经过并进行文献复习。

**结果:**

患者，女，66岁，因发现血小板减少1 d入院，入院查体皮肤、巩膜黄染，意识清，但计算力下降。辅助检查：PLT 7×10^9^/L；外周血涂片破碎红细胞4％；肝功：谷丙转氨酶55.2 U/L，谷草转氨酶117.5 U/L；总胆红素142.7 µmol/L，直接胆红素64.6 µmol/L，间接胆红素78.1 µmol/L；乳酸脱氢酶2362 U/L；肌酐260.7 µmol/L；血管性血友病因子裂解蛋白酶（ADAMTS13）活性2.7％且抑制物阳性（1.12 BU），符合iTTP诊断标准。接受血浆置换及甲泼尼龙治疗后症状及辅助检查未好转，利妥昔单抗因严重的输液反应停用，改用奥妥珠单抗（1000 mg每周1次×2周）获得18个月的疾病缓解，未发生严重不良事件。

**结论:**

对于利妥昔单抗不耐受的iTTP患者，奥妥珠单抗是安全、有效的替代治疗选择。

## Abstract (en)

**Objective:**

To explore the safety and efficacy of Obinutuzumab in the treatment of immune thrombotic thrombocytopenic purpura.

**Methods:**

Data from a case of immune thrombotic thrombocytopenic purpura (iTTP) admitted to the Department of haematology of the First Affiliated Hospital of Harbin Medical University were evaluated retrospectively and a literature review was performed.

**Results:**

A 66-year-old female patient was admitted to our hospital for thrombocytopenia. On admission, physical examination showed that yellow skin and sclera. The patient was fully conscious but had a decreasing ability to calculate. Laboratory examination revealed a platelet count of 7×10^9^/L; liver function: alanine aminotransferase 55.2 U/L, azelaic aminotransferase 117.5 U/L; total bilirubin 142.7 µmol/L, direct bilirubin 64.6 µmol/L, indirect bilirubin 78.1 µmol/L; lactate dehydrogenase: 2362 U/L; creatinine: 260.7 µmol/L; peripheral blood smear showed 4％ fragmented erythrocytes; ADAMTS13 activity 2.7％ and positive inhibitor（1.12 BU）. The patient is treated with plasma exchange and glucocorticoids, but the patient's symptoms worsened. Rituximab was permanently discontinued for severe infusion reactions. Treatment with Obinutuzumab（1000 mg, qw×2 w）and she achieved a complete remission for 18 months. The treatment was well tolerated with no adverse events related to Obinutuzumab.

**Conclusion:**

For iTTP patients with rituximab intolerance, obinutuzumab is a safe and effective alternative treatment option.

免疫性血栓性血小板减少性紫癜（iTTP）是一种危及生命的血栓性微血管病，其特征是微血管病性溶血性贫血、血小板减少和微血管血栓引起的缺血性脏器损伤[1]。血栓性血小板减少性紫癜（TTP）的发病机制主要是由于血管性血友病因子（VWF）裂解蛋白酶（ADAMTS13）自身抗体生成，导致ADAMTS13严重缺乏，不能剪切大片段的血管性血友病因子，使其在血管中暴露，聚集血小板从而导致广泛的血栓形成[2]–[3]。抗CD20单克隆抗体是iTTP的重要治疗手段，其可以清除产生ADAMTS13自身抗体的B淋巴细胞[4]–[5]。奥妥珠单抗（Obinutuzumab）是人源化的Ⅱ型抗CD20单克隆抗体，体外实验证实其较利妥昔单抗可更强的靶向杀伤B淋巴细胞，已有报道其作为不耐受利妥昔单抗的iTTP患者的替代治疗手段[6]。目前关于iTTP患者接受奥妥珠单抗治疗的数据仍然有限。这里我们报告国内首例接受奥妥珠单抗治疗的iTTP患者的病例，并对奥妥珠单抗治疗iTTP进行文献复习。

## 病例资料

患者，女，66岁，因“发现血小板减少1天”于2023年3月8日入我科住院治疗。患者入院前1 d突发视物模糊，伴发热、乏力、心前区疼痛，当地医院血常规示PLT 7×10^9^/L。入院查体：皮肤、巩膜黄染，意识清，但计算力下降。入院后急检血常规：WBC 8.41×10^9^/L，RBC 4.05×10^12^/L，HGB 125 g/L，PLT 7×10^9^/L；肝功能：谷丙转氨酶55.2 U/L，谷草转氨酶117.5 U/L；总胆红素142.7 µmol/L，直接胆红素64.6 µmol/L，间接胆红素78.1 µmol/L；乳酸脱氢酶（LDH）2362 U/L；肌酐（Cr）260.7 µmol/L。外周血涂片可见4％破碎红细胞，初步诊断为TTP，转入血液肿瘤重症监护病房，给予甲泼尼龙1 mg·kg^−1^·d^−1^及血浆置换（2000 ml每日1次）治疗。实验室检查回报：凝血检查大致正常，自身抗体相关检查均阴性，肌钙蛋白I（TnI）3930.1 ng/L，肌酸激酶同工酶6.5 µg/L，B型利钠肽前体（proBNP）98.3 ng/L，染色体及免疫分型无异常，ADAMTS活性2.7％，ADAMTS13抑制物1.12 BU。确定诊断：iTTP、肝功能异常、肾功能不全、高胆红素血症、心肌损伤、感染性发热。加用利妥昔单抗375 mg/m^2^，输注10 min后患者出现高热、寒颤、呼吸困难、血压下降，考虑输液反应、过敏性休克可能性大，立即停止输注，给予吸氧、皮下注射0.1％肾上腺素0.5 ml后好转，后续未再应用利妥昔单抗。经上述积极治疗5 d（2023年3月13日）后，PLT 14×10^9^/L，LDH 1388 U/L；意识障碍加重，躁动，需进行镇静；无尿，Cr进行性增高至532.1 µmol/L；TnI 19659.6 ng/L，proBNP 11615.3 ng/L；符合难治性TTP诊断标准[7]。2023年3月14日起调整治疗方案：①控制原发病：继续每日1次血浆置换，调整甲泼尼龙为1000 mg/d×3 d冲击治疗，后改为1 mg·kg^−1^·d^−1^；奥妥珠单抗1000 mg每周1次；②保护肾脏功能，持续肾脏替代治疗（CRRT）；③硝酸异山梨酯注射液静脉滴注。

2023年3月16日复查PLT 53×10^9^/L，LDH 365 U/L，Cr 436 µmol/L。2023年3月20日PLT恢复至161×10^9^/L，LDH 271 U/L，Cr 385 µmol/L；TnI 1511 ng/L，proBNP 3299 ng/L。停止血浆置换，继续甲泼尼龙1 mg·kg^−1^·d^−1^、奥妥珠单抗1000 mg每周1次。2023年3月30日复查PLT 144×10^9^/L；LDH 229 U/L，Cr 121 µmol/L，TnI 86 ng/L，proBNP 346 ng/L；淋巴细胞亚群B细胞数为0；ADAMTS13活性53.5％，ADAMTS13抑制物0 BU。意识恢复，甲泼尼龙逐渐减停，至2024年10月26日最后一次随访，疾病未复发。

## 讨论及文献复习

目前，iTTP的一线治疗方案包括：①从循环中清除ADAMTS13自身抗体：血浆置换；②使用免疫抑制减少ADAMTS13自身抗体的产生：糖皮质激素、利妥昔单抗；③阻断VWF与血小板糖蛋白GPⅠb结合：卡普塞珠单抗（Caplacizumab）等[1],[4]–[5]。联合治疗方案后iTTP的总生存率已超过90％[8]–[10]。利妥昔单抗是人鼠嵌合型Ⅰ型抗CD20单克隆抗体，已广泛用于iTTP患者的日常治疗，但多达10％的患者可能有输液反应、过敏反应，甚至是急性或迟发性利妥昔单抗诱导的血清病[11]–[12]。

奥妥珠单抗是人源化的Ⅱ型抗CD20单克隆抗体，其在Fc段的糖基化修饰可增强其与B淋巴细胞表面CD20分子的亲和力，从而增强抗体依赖性细胞介导的细胞毒作用（ADCC）和抗体依赖性细胞介导的吞噬作用[13]–[16]。在体外研究中，奥妥珠单抗诱导的ADCC活性比利妥昔单抗高35～100倍[13],[16]。临床前研究显示奥妥珠单抗还可诱导直接致细胞死亡（DCD）作用和补体依赖细胞毒性（CDC）作用[14],[16]。临床研究已经证实奥妥珠单抗联合化疗可有效降低初治慢性淋巴细胞白血病和初治/复发难治滤泡性淋巴瘤疾病进展风险，改善预后[17]–[19]。已有国内外研究证实，奥妥珠单抗在温抗体型自身免疫性溶血性贫血和免疫性血小板减少患者中取得了很好的疗效，在脾切除术无效和利妥昔单抗耐药的患者中也取得了较好的疗效[20]。Robertz等[6]报道了两例因输注不良反应和血清病对利妥昔单抗不耐受的iTTP患者，接受奥妥珠单抗治疗后分别维持了12个月和18个月的完全缓解。英国TTP登记处记录了8例应用奥妥珠单抗治疗的TTP患者，所有患者均达到疾病缓解（ADAMTS13活性≥30 IU/dl，中位缓解时间15 d），中位无复发生存期为17.4个月[18]。这项结果表明，对于iTTP患者奥妥珠单抗可作为不耐受利妥昔单抗治疗的替代选择，并具有良好的安全性和疗效[21]–[22]。

本例患者急性起病，以突发视物模糊为首要表现，伴有发热，计算力下降，血小板减少，肝肾功异常，胆红素增高，外周血涂片可见破碎红细胞，TTP可能性极大，在送检ADAMTS13活性和抑制物同时启用血浆置换及糖皮质激素治疗。结果回报符合iTTP诊断，加用利妥昔单抗375 mg/kg，因严重输液反应停用。在血浆置换及甲泼尼龙治疗情况下病情持续进展，多脏器功能损伤加重，考虑为难治性TTP。因此继续血浆置换的基础上，调整治疗为甲泼尼龙冲击治疗联合奥妥珠单抗1000 mg每周1次，同时加强支持治疗。联合治疗后患者症状及化验指标好转，淋巴细胞亚群检测示B淋巴细胞数量下降至0，ADAMTS13活性恢复且抑制物转阴，随访18个月无疾病复发，未发生不良反应。

综上所述，对于iTTP患者而言，抗CD20单克隆抗体可通过清除B淋巴细胞，在根源上阻止ADAMTS13自身抗体的产生，是重要的治疗手段。因输液反应、过敏反应，甚至是急性或迟发性血清病不能耐受利妥昔单抗的患者，奥妥珠单抗可作为替代选择。奥妥珠单抗治疗iTTP的合理用药周期、疗效和安全性尚需进一步研究。
